# *N*-(3,5-Dimethyl­phen­yl)-4-methyl­benzamide

**DOI:** 10.1107/S1600536811044904

**Published:** 2011-11-02

**Authors:** Vinola Z. Rodrigues, Peter Herich, B. Thimme Gowda, Jozef Kožíšek

**Affiliations:** a Department of Chemistry, Mangalore University, Mangalagangotri 574 199, Mangalore, India; b Institute of Physical Chemistry and Chemical Physics, Slovak University of Technology, Radlinského 9, SK-812 37 Bratislava, Slovak Republic

## Abstract

In the title compound, C_16_H_17_NO, the dihedral angle between the two benzene rings is 16.6 (1)°. The crystal structure is stabilized by inter­molecular N—H⋯O hydrogen bonds, which link the mol­ecules into chains running along the *c* axis.

## Related literature

For the preparation of the title compound, see: Gowda *et al.* (2003 ▶). For our studies on the effects of substituents on the structures and other aspects of *N*-(ar­yl)-amides, see: Bhat & Gowda (2000 ▶); Bowes *et al.* (2003 ▶); Gowda *et al.* (2009 ▶); Saeed *et al.* (2010 ▶), on *N*-(ar­yl)-methane­sulfonamides, see: Gowda *et al.* (2007 ▶), on *N*-(ar­yl)-aryl­sulfonamides, see: Shetty & Gowda (2005 ▶) and on *N*-chloro-aryl­amides, see: Gowda & Weiss (1994 ▶).
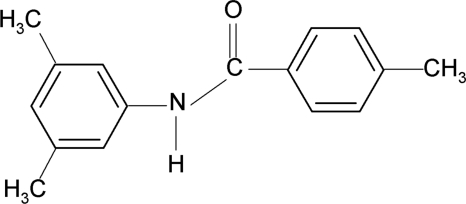


## Experimental

### 

#### Crystal data

C_16_H_17_NO
*M* *_r_* = 239.31
Monoclinic, 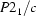

*a* = 15.9048 (6) Å
*b* = 9.0323 (4) Å
*c* = 9.6774 (3) Å
β = 93.619 (3)°
*V* = 1387.45 (9) Å^3^
*Z* = 4
Mo *K*α radiation
μ = 0.07 mm^−1^
*T* = 293 K
0.85 × 0.22 × 0.10 mm

#### Data collection

Oxford Diffraction Xcalibur Ruby Gemini diffractometer
Absorption correction: analytical [*CrysAlis RED* (Oxford Diffraction, 2009 ▶) based on expressions derived (Clark & Reid, 1995 ▶)] *T*_min_ = 0.981, *T*_max_ = 0.993
24108 measured reflections
3853 independent reflections
1720 reflections with *I* > 2σ(*I*)
*R*_int_ = 0.029

#### Refinement

*R*[*F* ^2^ > 2σ(*F* ^2^)] = 0.045
*wR*(*F* ^2^) = 0.143
*S* = 0.95
3853 reflections
160 parameters
H-atom parameters constrained
Δρ_max_ = 0.16 e Å^−3^
Δρ_min_ = −0.17 e Å^−3^

### 

Data collection: *CrysAlis CCD* (Oxford Diffraction, 2009 ▶); cell refinement: *CrysAlis CCD*; data reduction: *CrysAlis RED* (Oxford Diffraction, 2009 ▶); program(s) used to solve structure: *SHELXS97* (Sheldrick, 2008 ▶); program(s) used to refine structure: *SHELXL97* (Sheldrick, 2008 ▶); molecular graphics: *DIAMOND* (Brandenburg, 2002 ▶); software used to prepare material for publication: *enCIFer* (Allen *et al.*, 2004 ▶).

## Supplementary Material

Crystal structure: contains datablock(s) I, global. DOI: 10.1107/S1600536811044904/bt5690sup1.cif

Structure factors: contains datablock(s) I. DOI: 10.1107/S1600536811044904/bt5690Isup2.hkl

Supplementary material file. DOI: 10.1107/S1600536811044904/bt5690Isup3.cml

Additional supplementary materials:  crystallographic information; 3D view; checkCIF report

## Figures and Tables

**Table 1:** Hydrogen-bond geometry (Å, °)

*D*—H⋯*A*	*D*—H	H⋯*A*	*D*⋯*A*	*D*—H⋯*A*
N1—H1*A*⋯O1^i^	0.86	2.16	2.9379 (13)	151

Symmetry code: (i) 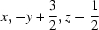.

**Fig. 1.**
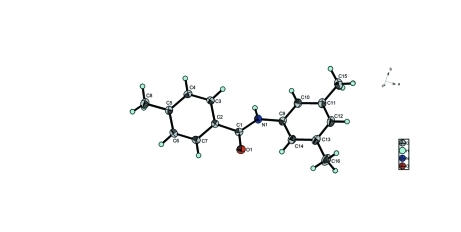
Molecular structure of the title compound showing the atom labelling scheme. Displacement ellipsoids are drawn at the 50% probability level. H atoms are represented as small spheres of arbitrary radii.

**Fig. 2.**
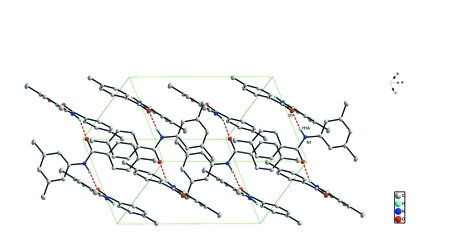
Part of the crystal structure of the title compound. Molecular chains are generated by N—H···O hydrogen bonds which are shown by dashed lines. H atoms not involved in intermolecular bonding have been omitted.

## supplementary crystallographic information

### Comment

The amide and sulfonamide moieties are the constituents of many biologically important compounds. As part of our work on the substituent effects on the structures and other aspects of *N*-(aryl)-amides (Bhat & Gowda, 2000; Bowes *et al.*, 2003; Gowda *et al.*, 2009; Saeed *et al.*, 2010, *N*-(aryl)-methanesulfonamides (Gowda *et al.*, 2007), *N*-(aryl)-arylsulfonamides (Shetty & Gowda, 2005) and *N*-chloro-arylamides (Gowda & Weiss, 1994), in the present work, the crystal structure of *N*-(3,5-Dimethylphenyl)-4-methylbenzamide (I) has been determined (Fig.1).

In (I), the conformation of the the N–H bond is positioned *syn* to one of the *meta*-methyl groups in the anilino ring and *anti* to the other *meta*-methyl group. Further, the N—H and C=O bonds in the C—NH—C(O)—C segment are *anti* to each other, similar to that observed in *N*-(2,6-dimethylphenyl)-4-methylbenzamide (II) (Gowda *et al.*, 2009).

The –C–NH–C(=O)–C– is almost linear with the torsional angle of 174.4 (1)°. Further, the dihedral angle between the two benzene rings is 16.6 (1)°, compared to the value of 78.8 (1)° in (II).

The packing of molecules linked by N—H···O hydrogen bonds into infinite chains is shown in Fig. 2.

### Experimental

The title compound was prepared according to the method described by Gowda *et al.* (2003). The purity of the compound was checked by determining its melting point. It was characterized by recording its infrared and NMR spectra. Rod-like colourless single crystals of the title compound were obtained by slow evaporation from an ethanol solution of the compound (0.5 g in about 30 ml of ethanol) at room temperature.

### Refinement

All H atoms were visible in difference maps and then treated as riding atoms with C–H distances of 0.93Å (C-aromatic), 0.96Å (C-methyl) and N—H = 0.86 Å. The *U*_iso_(H) values were set at 1.2 *U*_eq_(C-aromatic, N) and 1.5 *U*_eq_(C-methyl).

### Crystal data

**Table:**

C_16_H_17_NO	*F*(000) = 512
*M**_r_* = 239.31	*D*_x_ = 1.146 Mg m^−^^3^
Monoclinic, *P*2_1_/*c*	Mo *K*α radiation, λ = 0.71073 Å
Hall symbol: -P 2ybc	Cell parameters from 9180 reflections
*a* = 15.9048 (6) Å	θ = 3.4–29.5°
*b* = 9.0323 (4) Å	µ = 0.07 mm^−^^1^
*c* = 9.6774 (3) Å	*T* = 293 K
β = 93.619 (3)°	Rod, colorless
*V* = 1387.45 (9) Å^3^	0.85 × 0.22 × 0.10 mm
*Z* = 4	

### Data collection

**Table:**

Oxford Diffraction Xcalibur Ruby Gemini diffractometer	3853 independent reflections
Radiation source: Enhance (Mo) X-ray Source	1720 reflections with *I* > 2σ(*I*)
graphite	*R*_int_ = 0.029
Detector resolution: 10.4340 pixels mm^-1^	θ_max_ = 29.5°, θ_min_ = 3.4°
ω scans	*h* = −22→22
Absorption correction: analytical [*CrysAlis RED* (Oxford Diffraction, 2009) based on expressions derived (Clark & Reid, 1995)]	*k* = −12→12
*T*_min_ = 0.981, *T*_max_ = 0.993	*l* = −13→13
24108 measured reflections	

### Refinement

**Table:**

Refinement on *F*^2^	Primary atom site location: structure-invariant direct methods
Least-squares matrix: full	Secondary atom site location: difference Fourier map
*R*[*F*^2^ > 2σ(*F*^2^)] = 0.045	Hydrogen site location: inferred from neighbouring sites
*wR*(*F*^2^) = 0.143	H-atom parameters constrained
*S* = 0.95	*w* = 1/[σ^2^(*F*_o_^2^) + (0.0807*P*)^2^] where *P* = (*F*_o_^2^ + 2*F*_c_^2^)/3
3853 reflections	(Δ/σ)_max_ < 0.001
160 parameters	Δρ_max_ = 0.16 e Å^−^^3^
0 restraints	Δρ_min_ = −0.17 e Å^−^^3^

### Special details

**Table:**

Experimental. CrysAlis RED (Oxford Diffraction, 2009) Analytical numeric absorption correction using a multifaceted crystal model based on expressions derived (Clark & Reid, 1995).
Geometry. All e.s.d.'s (except the e.s.d. in the dihedral angle between two l.s. planes) are estimated using the full covariance matrix. The cell e.s.d.'s are taken into account individually in the estimation of e.s.d.'s in distances, angles and torsion angles; correlations between e.s.d.'s in cell parameters are only used when they are defined by crystal symmetry. An approximate (isotropic) treatment of cell e.s.d.'s is used for estimating e.s.d.'s involving l.s. planes.
Refinement. Refinement of *F*^2^ against ALL reflections. The weighted *R*-factor *wR* and goodness of fit *S* are based on *F*^2^, conventional *R*-factors *R* are based on *F*, with *F* set to zero for negative *F*^2^. The threshold expression of *F*^2^ > σ(*F*^2^) is used only for calculating *R*-factors(gt) *etc*. and is not relevant to the choice of reflections for refinement. *R*-factors based on *F*^2^ are statistically about twice as large as those based on *F*, and *R*- factors based on ALL data will be even larger.

### Fractional atomic coordinates and isotropic or equivalent isotropic displacement parameters (Å^2^)

**Table:**

	*x*	*y*	*z*	*U*_iso_*/*U*_eq_	
C1	0.10384 (9)	0.72822 (17)	0.65724 (13)	0.0516 (4)	
C2	0.01648 (9)	0.69750 (16)	0.60172 (12)	0.0490 (4)	
C3	−0.02569 (10)	0.78306 (18)	0.50007 (14)	0.0597 (4)	
H3A	0.0010	0.8648	0.4641	0.072*	
C4	−0.10664 (10)	0.7479 (2)	0.45208 (15)	0.0662 (5)	
H4A	−0.1335	0.8063	0.3835	0.079*	
C5	−0.14900 (9)	0.62795 (19)	0.50320 (14)	0.0589 (4)	
C6	−0.10688 (10)	0.54526 (18)	0.60578 (13)	0.0590 (4)	
H6A	−0.1340	0.4647	0.6430	0.071*	
C7	−0.02621 (10)	0.57839 (17)	0.65440 (12)	0.0558 (4)	
H7A	0.0002	0.5202	0.7236	0.067*	
C8	−0.23550 (11)	0.5858 (2)	0.44574 (19)	0.0857 (6)	
H8C	−0.2670	0.5457	0.5184	0.103*	
H8B	−0.2311	0.5128	0.3744	0.103*	
H8A	−0.2638	0.6719	0.4076	0.103*	
C9	0.23454 (9)	0.86647 (18)	0.60272 (13)	0.0554 (4)	
C10	0.26402 (10)	0.96307 (18)	0.50518 (14)	0.0620 (4)	
H10A	0.2297	0.9857	0.4266	0.074*	
C11	0.34300 (10)	1.0264 (2)	0.52210 (16)	0.0705 (5)	
C12	0.39208 (11)	0.9929 (2)	0.64150 (19)	0.0800 (5)	
H12A	0.4448	1.0367	0.6557	0.096*	
C13	0.36468 (11)	0.8959 (2)	0.74052 (16)	0.0737 (5)	
C14	0.28568 (10)	0.8325 (2)	0.72012 (14)	0.0652 (5)	
H14A	0.2667	0.7669	0.7853	0.078*	
C15	0.37495 (9)	1.12687 (19)	0.41307 (14)	0.1005 (7)	
H15C	0.4201	1.1866	0.4531	0.121*	
H15B	0.3300	1.1898	0.3773	0.121*	
H15A	0.3951	1.0684	0.3392	0.121*	
C16	0.41977 (9)	0.86018 (19)	0.86910 (14)	0.1075 (8)	
H16C	0.3865	0.8133	0.9360	0.129*	
H16B	0.4437	0.9499	0.9075	0.129*	
H16A	0.4642	0.7946	0.8456	0.129*	
N1	0.15225 (8)	0.80933 (14)	0.57539 (11)	0.0572 (4)	
H1A	0.1296	0.8292	0.4945	0.069*	
O1	0.12997 (7)	0.68252 (12)	0.77180 (8)	0.0649 (3)	

### Atomic displacement parameters (Å^2^)

**Table:**

	*U*^11^	*U*^22^	*U*^33^	*U*^12^	*U*^13^	*U*^23^
C1	0.0602 (9)	0.0549 (9)	0.0394 (7)	0.0012 (7)	0.0015 (6)	−0.0079 (6)
C2	0.0573 (9)	0.0507 (9)	0.0391 (7)	−0.0011 (7)	0.0039 (6)	−0.0056 (6)
C3	0.0602 (10)	0.0559 (10)	0.0628 (8)	−0.0039 (8)	0.0028 (7)	0.0083 (7)
C4	0.0598 (11)	0.0686 (11)	0.0693 (9)	0.0056 (9)	−0.0036 (8)	0.0113 (8)
C5	0.0544 (10)	0.0634 (11)	0.0592 (8)	−0.0015 (8)	0.0053 (7)	−0.0083 (7)
C6	0.0674 (10)	0.0561 (10)	0.0542 (8)	−0.0110 (8)	0.0095 (7)	−0.0034 (7)
C7	0.0688 (10)	0.0558 (10)	0.0424 (7)	−0.0017 (8)	0.0013 (6)	−0.0001 (6)
C8	0.0639 (12)	0.0939 (15)	0.0983 (12)	−0.0037 (10)	−0.0028 (9)	−0.0059 (11)
C9	0.0508 (9)	0.0620 (10)	0.0531 (8)	−0.0002 (7)	0.0005 (6)	−0.0116 (7)
C10	0.0567 (10)	0.0700 (11)	0.0592 (8)	−0.0028 (8)	0.0030 (7)	−0.0077 (7)
C11	0.0590 (11)	0.0728 (12)	0.0800 (10)	−0.0053 (9)	0.0074 (8)	−0.0072 (9)
C12	0.0520 (10)	0.0856 (14)	0.1017 (13)	−0.0087 (10)	−0.0009 (9)	−0.0170 (11)
C13	0.0562 (11)	0.0865 (14)	0.0767 (10)	0.0031 (9)	−0.0097 (8)	−0.0090 (9)
C14	0.0578 (10)	0.0753 (12)	0.0613 (9)	−0.0011 (8)	−0.0050 (7)	−0.0032 (7)
C15	0.0800 (14)	0.1071 (18)	0.1156 (14)	−0.0235 (12)	0.0155 (11)	0.0103 (13)
C16	0.0702 (13)	0.141 (2)	0.1072 (14)	−0.0027 (13)	−0.0314 (11)	0.0019 (13)
N1	0.0568 (8)	0.0726 (9)	0.0415 (6)	−0.0092 (7)	−0.0036 (5)	0.0006 (5)
O1	0.0700 (7)	0.0809 (8)	0.0428 (6)	−0.0007 (6)	−0.0041 (5)	0.0038 (5)

### Geometric parameters (Å, °)

**Table:**

C1—O1	1.2305 (15)	C9—C14	1.389 (2)
C1—N1	1.3542 (18)	C9—N1	1.4162 (18)
C1—C2	1.484 (2)	C10—C11	1.380 (2)
C2—C7	1.386 (2)	C10—H10A	0.9300
C2—C3	1.3898 (19)	C11—C12	1.386 (2)
C3—C4	1.378 (2)	C11—C15	1.504 (2)
C3—H3A	0.9300	C12—C13	1.389 (2)
C4—C5	1.384 (2)	C12—H12A	0.9300
C4—H4A	0.9300	C13—C14	1.383 (2)
C5—C6	1.381 (2)	C13—C16	1.511 (2)
C5—C8	1.500 (2)	C14—H14A	0.9300
C6—C7	1.372 (2)	C15—H15C	0.9600
C6—H6A	0.9300	C15—H15B	0.9600
C7—H7A	0.9300	C15—H15A	0.9600
C8—H8C	0.9600	C16—H16C	0.9600
C8—H8B	0.9600	C16—H16B	0.9600
C8—H8A	0.9600	C16—H16A	0.9600
C9—C10	1.388 (2)	N1—H1A	0.8600
			
O1—C1—N1	122.44 (13)	C11—C10—C9	121.56 (14)
O1—C1—C2	121.25 (13)	C11—C10—H10A	119.2
N1—C1—C2	116.32 (12)	C9—C10—H10A	119.2
C7—C2—C3	117.78 (14)	C10—C11—C12	117.97 (16)
C7—C2—C1	118.76 (13)	C10—C11—C15	120.79 (14)
C3—C2—C1	123.46 (13)	C12—C11—C15	121.24 (15)
C4—C3—C2	120.62 (15)	C11—C12—C13	121.86 (16)
C4—C3—H3A	119.7	C11—C12—H12A	119.1
C2—C3—H3A	119.7	C13—C12—H12A	119.1
C3—C4—C5	121.71 (14)	C14—C13—C12	118.96 (15)
C3—C4—H4A	119.1	C14—C13—C16	120.24 (16)
C5—C4—H4A	119.1	C12—C13—C16	120.80 (15)
C6—C5—C4	117.08 (14)	C13—C14—C9	120.34 (16)
C6—C5—C8	121.40 (16)	C13—C14—H14A	119.8
C4—C5—C8	121.47 (15)	C9—C14—H14A	119.8
C7—C6—C5	121.96 (15)	C11—C15—H15C	109.5
C7—C6—H6A	119.0	C11—C15—H15B	109.5
C5—C6—H6A	119.0	H15C—C15—H15B	109.5
C6—C7—C2	120.83 (14)	C11—C15—H15A	109.5
C6—C7—H7A	119.6	H15C—C15—H15A	109.5
C2—C7—H7A	119.6	H15B—C15—H15A	109.5
C5—C8—H8C	109.5	C13—C16—H16C	109.5
C5—C8—H8B	109.5	C13—C16—H16B	109.5
H8C—C8—H8B	109.5	H16C—C16—H16B	109.5
C5—C8—H8A	109.5	C13—C16—H16A	109.5
H8C—C8—H8A	109.5	H16C—C16—H16A	109.5
H8B—C8—H8A	109.5	H16B—C16—H16A	109.5
C10—C9—C14	119.29 (14)	C1—N1—C9	129.85 (12)
C10—C9—N1	116.73 (12)	C1—N1—H1A	115.1
C14—C9—N1	123.98 (14)	C9—N1—H1A	115.1
			
O1—C1—C2—C7	−21.8 (2)	N1—C9—C10—C11	179.38 (14)
N1—C1—C2—C7	158.54 (12)	C9—C10—C11—C12	−1.2 (2)
O1—C1—C2—C3	157.45 (13)	C9—C10—C11—C15	177.99 (14)
N1—C1—C2—C3	−22.2 (2)	C10—C11—C12—C13	1.7 (3)
C7—C2—C3—C4	−1.3 (2)	C15—C11—C12—C13	−177.49 (17)
C1—C2—C3—C4	179.48 (13)	C11—C12—C13—C14	−0.9 (3)
C2—C3—C4—C5	0.5 (2)	C11—C12—C13—C16	179.40 (16)
C3—C4—C5—C6	0.6 (2)	C12—C13—C14—C9	−0.4 (3)
C3—C4—C5—C8	−176.97 (15)	C16—C13—C14—C9	179.28 (14)
C4—C5—C6—C7	−0.8 (2)	C10—C9—C14—C13	0.9 (2)
C8—C5—C6—C7	176.75 (14)	N1—C9—C14—C13	−178.51 (14)
C5—C6—C7—C2	0.0 (2)	O1—C1—N1—C9	−5.2 (2)
C3—C2—C7—C6	1.0 (2)	C2—C1—N1—C9	174.40 (13)
C1—C2—C7—C6	−179.66 (12)	C10—C9—N1—C1	−171.53 (14)
C14—C9—C10—C11	0.0 (2)	C14—C9—N1—C1	7.9 (2)

### Hydrogen-bond geometry (Å, °)

**Table:**

*D*—H···*A*	*D*—H	H···*A*	*D*···*A*	*D*—H···*A*
N1—H1A···O1^i^	0.86	2.16	2.9379 (13)	151.

Symmetry codes: (i) *x*, −*y*+3/2, *z*−1/2.
